# Small-molecule RL71-triggered excessive autophagic cell death as a potential therapeutic strategy in triple-negative breast cancer

**DOI:** 10.1038/cddis.2017.444

**Published:** 2017-09-14

**Authors:** Jian Gao, Minmin Fan, Shuang Peng, Minxia Zhang, Gang Xiang, Xin Li, Wenjie Guo, Yang Sun, Xuefeng Wu, Xudong Wu, Guang Liang, Yan Shen, Qiang Xu

**Affiliations:** 1 State Key Laboratory of Pharmaceutical Biotechnology, School of Life Sciences, Nanjing University, Nanjing 210093, China; 2 Bioorganic and Medicinal Chemistry Research Center, School of Pharmaceutical Sciences, Wenzhou Medical College, Wenzhou 325035, China; 3 Collaborative Innovation Center of Chemistry for Life Sciences, Nanjing University, Nanjing 210093, China

## Abstract

Triple-negative breast cancer (TNBC) has an aggressive phenotype and a poor prognosis owing to the high propensity for metastatic progression and the absence of specific targeted treatment. Here, we revealed that small-molecule RL71 targeting sarco/endoplasmic reticulum calcium-ATPase 2 (SERCA2) exhibited potent anti-cancer activity on all TNBC cells tested. Apart from apoptosis induction, RL71 triggered excessive autophagic cell death, the main contributor to RL71-induced TNBC cell death. RL71 augmented the release of Ca^2+^ from the endoplasmic reticulum (ER) into the cytosol by inhibiting SERCA2 activity. The disruption of calcium homeostasis induced ER stress, leading to apoptosis. More importantly, the elevated intracellular calcium signals induced autophagy through the activation of the CaMKK-AMPK-mTOR pathway and mitochondrial damage. In two TNBC xenograft mouse models, RL71 also displayed strong efficacy including the inhibition of tumor growth, the reduction of metastasis, as well as the prolongation of survival time. These findings suggest SERCA2 as a previous unknown target candidate for TNBC treatment and support the idea that autophagy inducers could be useful as new therapeutics in TNBC treatment.

Triple-negative breast cancer (TNBC), defined by a lack of expression of estrogen receptor and progesterone receptor as well as expression or amplification of human epidermal growth factor receptor 2, accounts for 15–20% of all breast cancers.^1, 2^ To date, chemotherapy remains the standard therapeutic approach for TNBC at all stages. TNBC is initially sensitive to standard chemotherapy, but has a high rate of local recurrence and systemic metastasis that are unresponsive to current therapies. The lack of targeted therapies and the poor disease prognosis have fostered a major effort to discover potential molecular targets to treat patients with TNBC.

Autophagy is a catabolic process that delivers cellular components such as cytosolic protein aggregates and excessive or defective organelles for degradation and recycling in the lysosome.^3, 4^ It can be activated by stressful conditions such as nutrient starvation, oxidative stress and endoplasmic reticulum (ER) stress. Despite its involvement in a survival mechanism, excessive activation of autophagy may eventually lead to type II programmed cell death in cancers.^5, 6^ A number of clinically approved drugs or experimental small-molecule compounds have been shown to induce autophagic cell death, which is responsible for their potential antitumor activities.^7, 8, 9, 10^ Induction of autophagic cell death may provide an alternative therapeutic approach for cancer therapy except for apoptosis induction. Recent studies demonstrate that the expression of autophagy-related markers LC3 and Beclin-1 in TNBC subtype was the highest among breast cancers, suggesting constitutive activation of autophagy in TNBC.^11^ Given the threshold effect of autophagy distinguishing survival and death in cancer cells, we hypothesized that further facilitating autophagy with a small-molecule inducer may be beneficial for the development of a novel therapeutic strategy for TNBC.

We and other colleagues have previously shown that RL71, a second-generation curcumin analog, exhibits potent cytotoxicity towards a variety of human cancer cells, including TNBC cell lines MDA-MB-231 and MDA-MB-468.^12, 13, 14^ Recently, we have identified sarco/endoplasmic reticulum calcium-ATPase 2 (SERCA2) as the direct target of RL71 that inhibits its Ca^2+^-ATPase activity and leads to ER stress-associated apoptosis.^14^ However, <20% of MDA-MB-468 cells underwent apoptosis 48 h following RL71 treatment (1 *μ*M), which seems insufficient to account for its sub micromolar IC_50_ value.^12^ In this study, we demonstrate that apart from apoptosis, excessive autophagy mediated RL71-induced TNBC cell death *in vitro* and *in vivo*, and small-molecule-triggered excessive autophagic cell death through targeting SERCA2 may be a potential therapeutic strategy for TNBC.

## Results

### RL71 shows potent cytotoxicity towards TNBC cell lines mainly through the induction of autophagic cell death

Similar to the previous study,^12^ RL71 exhibited a deleterious effect on three different TNBC cell lines including PTEN-null MDA-MB-468, KRAS-mutant MDA-MB-231 and BRCA1-mutant SUM-1315. MTT assay showed that exposure to RL71 led to a dose- and time- dependent decrease in cell viability (Supplementary Figure S1a and b). The 50% inhibitory concentrations (IC_50_) in all three cell lines were about 1 *μ*M of RL71 after 48 h of treatment (Supplementary Figure S1c). Trypan Blue dye exclusion method also showed the reduction of viable cell numbers (Supplementary Figure S1d). Typical chromatin condensation and nuclei blebbing were observed in some RL71-treated cells after Hoechst staining, indicative of apoptosis induction (Supplementary Figure S1e). However, the percentages of apoptotic cells were <10% in all three TNBC cell lines after 24 h treatment with 1 *μ*M of RL71. When the cells were pretreated with a pan-caspase inhibitor (zVAD-FMK, zVAD), its inhibitory effect on cell viability was mildly reversed by increasing survival rates (MDA-MB-468, from 47 to 65% MDA-MB-231, from 65 to 76% SUM-1315, from 56 to 69% Supplementary Figure S1f). Necrostatin-1, a necroptosis inhibitor, failed to attenuate the effect of RL71 on TNBC cells (data not shown). In addition, annexin V/PI double staining showed that RL71-induced apoptosis was abolished by zVAD (Supplementary Figure S1g). These data suggest that other mechanisms could possibly account for the RL71-mediated cytotoxicity rather than caspase-dependent apoptosis or necrosis induction.

**Figure 1:**
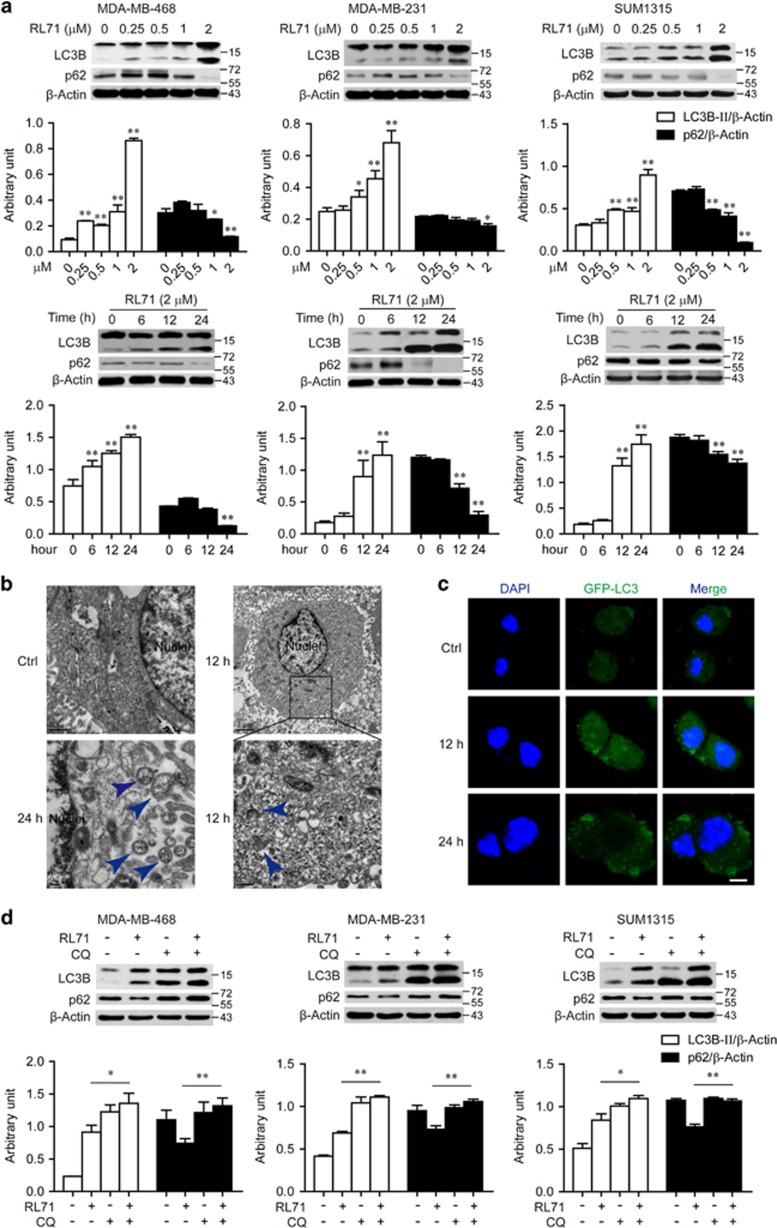
RL71 induces autophagy in TNBC cell lines. (**a**) Indicated TNBC cell lines were incubated with various concentrations of RL71 (0–2 *μ*M) for 24 h or in the presence of RL71 (2 *μ*M) for different time courses. The protein levels of LC3B and p62 were determined by western blot from whole lysates. *β*-Actin was used as a loading control. (**b**) MDA-MB-468 cells were treated with RL71 (2 *μ*M) for 12 or 24 h. Electron microscopy images present the ultrastructure in the cells. Blue arrow, autophagic vaculos with distinct double membrane. (**c**) MDA-MB-468 cells were transiently transfected with the GFP-LC3 plasmid for 24 h and then treated with RL71 (2 *μ*M) for 12 or 24 h. Representative images show GFP-LC3 localization. Scale bar: 5 *μ*m. (**d**) Cells were treated with RL71 (1 *μ*M) or CQ (20 *μ*M) alone or in combination for 24 h. The protein levels of LC3B and p62 were determined by western blot. *P<0.05, **P<0.01

To test the role of autophagy in RL71-mediated cytotoxicity, we measured by western blot the lipidation of autophagy-related LC3B protein and the expression of p62 that is selectively incorporated into the autophagosome and degraded upon autophagy induction.^15^ The LC3B-I to LC3B-II-conversion was enhanced, whereas the p62 protein levels were reduced by RL71 in dose- and time-dependent manner in all three TNBC cell lines (Figure 1a). MDA-MB-468 cells were chosen for more detailed study since they showed strongest response to RL71 cytotoxicity. Upon RL71 treatment, electron microscopy images showed typical signs of autophagy, including accumulation of numerous vesicles with a distinct double membrane (Figure 1b). A time-dependent accumulation of such autophagic vaculos was observed starting at 12 h of RL71 treatment. Consistently, the formation of green fluorescent protein (GFP)-fused LC3 puncta was increased within MDA-MB-468 cells in response to RL71 treatment (Figure 1c). When combined with autophagolysosome fusion inhibitor CQ, RL71 was able to significantly enhance the LC3B-II levels compared with its sole treatment in all the three TNBC cell lines (Figure 1d), supporting autophagy induction.

**Figure 2:**
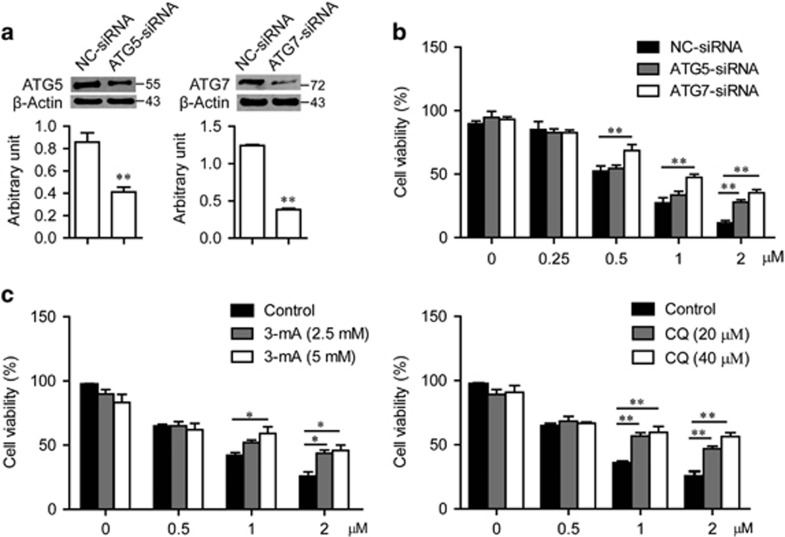
RL71-induced cell death is mainly mediated by autophagy. MDA-MB-468 cells were transiently transfected with control siRNA, siRNA targeting *ATG5* or *ATG7* for 24 h, and then treated with various concentrations of RL71 for 48 h. (**a**) Knockdown of *ATG5* or *ATG7* was confirmed by western blot. **P<0.01 *versus* NC-siRNA controls. (**b**) Cell viability was determined by MTT assay. (**c**) MDA-MB-468 cells were pretreated with 3-MA or CQ for 2 h before treatment with various concentrations of RL71 for 24 h. Cell viability was determined by MTT assay. The data are the mean±S.D. of three independent experiments. *P<0.05, **P<0.01, ***P<0.005

To clarify whether RL71-induced cell death is mediated through autophagy, we suppressed autophagy by knocking down the autophagy-related gene *ATG5* or *ATG7* (Figure 2a). Cell death induced by RL71 was remarkably reduced in the resulting MDA-MB-468 cells (Figure 2b). Furthermore, pretreatment with autophagy inhibitor 3-MA or CQ reversed RL71-induced cell death in a dose-dependent manner in MDA-MB-468 cells (Figure 2c). Taken together, these data suggest that autophagic cell death could be the main contributor to RL71-induced TNBC cell death.

### RL71 modulates intracellular calcium signaling by inhibiting SERCA2, leading to ER stress

**Figure 3:**
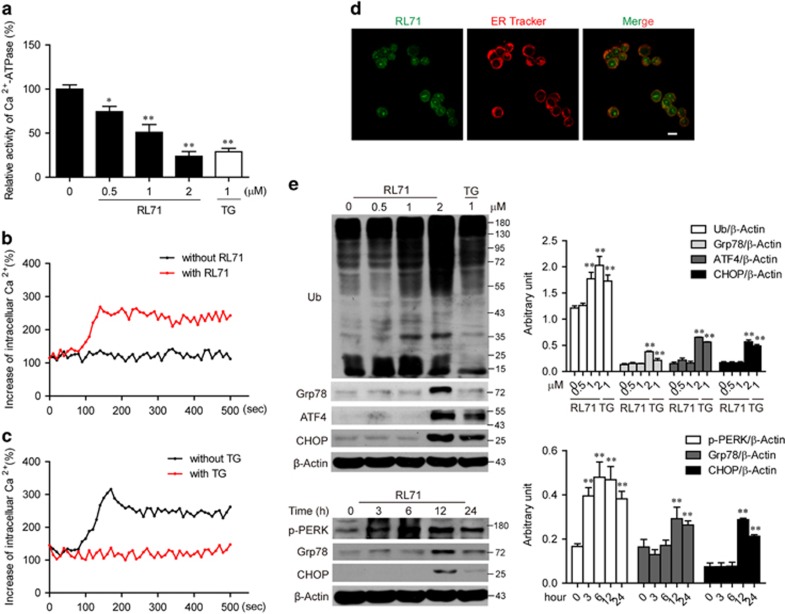
RL71 induces ER stress by targeting SERCA. (**a**) MDA-MB-468 cells were treated with various concentrations of RL71 or TG (1 *μ*M) for 24 h. Then, the Ca^2+^-ATPase activity was measured according to the instruction. *P<0.05, **P<0.01 *versus* untreated controls. (**b**) Fura-2/AM loaded MDA-MB-468 cells were stimulated with or without RL71 (2 *μ*M). The *y* axes represent the percentage of intracellular Ca^2+^ concentration. The *x* axes depict the time in seconds, with time 0 representing the time of RL71 addition. The data are representative of at least 3 experiments. (**c**) Changes of [Ca^2+^]_i_ after pretreatment with or without TG (5 *μ*M), followed by stimulation with RL71 (2 *μ*M). The data are representative of at least three experiments. (**d**) MDA-MB-468 cells were treated with 10 *μ*M of RL71 for 2 h and stained with ER tracker. Confocal microscopy was performed after a 2 h incubation. Scale bar: 10 *μ*m. (**e**) MDA-MB-468 were incubated with various concentrations of RL71 or TG (1 *μ*M) for 24 h or in the presence of RL71 (2 *μ*M) for different time courses. The protein levels of ubiquitin-linked proteins (Ub), Grp78, ATF4, CHOP and p-PERK were determined by western blot. *β*-Actin was used as a loading control. **P<0.01 *versus* untreated controls

As our previous study identified RL71 as a novel SERCA2 inhibitor that regulates intracellular calcium signaling,^14^ we measured Ca^2+^-ATPase activity in the treated MDA-MB-468 cells. As shown in Figure 3a, RL71 significantly inhibited the Ca^2+^-ATPase activity in a dose-dependent manner. A total of 2 *μ*M of RL71 inhibited the Ca^2+^-ATPase activity to a level comparable to 1 *μ*M of the pan-SERCA inhibitor thapsigargin (TG). Because SERCA-dependent calcium transport is the only calcium-uptake mechanism in the ER,^16^ the intracellular Ca^2+^ mobilization was examined in the treated MDA-MB-468 cells. In Ca^2+^-free medium, RL71 induced a steady rise in cytosolic Ca^2+^ levels (Figure 3b). When the cells were pretreated with TG that depletes Ca^2+^ stores in ER, RL71-induced [Ca^2+^]_i_ increase was abolished (Figure 3c). These data suggest that the cytosolic influx is due to SERCA inhibition in ER, but not extracellular calcium influx. To further confirm the SERCA2-targeting action of RL71 in the ER, we analyzed the localization of RL71 using its innate fluorescent property and subsequent confocal analysis. We observed a co-localization between RL71 and ER Tracker in MDA-MB-468 cells (Figure 3d). Knockdown of SERCA2 significantly reversed RL71-induced cell death in MDA-MB-468 cells (Supplementary Figure S2).

A depletion of the ER calcium storage pool and a subsequent rise in cytosolic calcium levels are generally associated with ER stress.^17^ We thus examined the key players of ER stress by western blot on the lysate of the treated MDA-MB-468 cells. RL71 treatment caused the accumulation of ubiquitinated proteins in a dose-dependent manner (Figure 3e). The expression of Grp78, ATF4 and CHOP had strongly increased by RL71. The increase in the expression of phosphorylated PERK, Grp78 and CHOP reached the plaque after 12 h of treatment.

### RL71-induced autophagy is independent of ER stress

**Figure 4:**
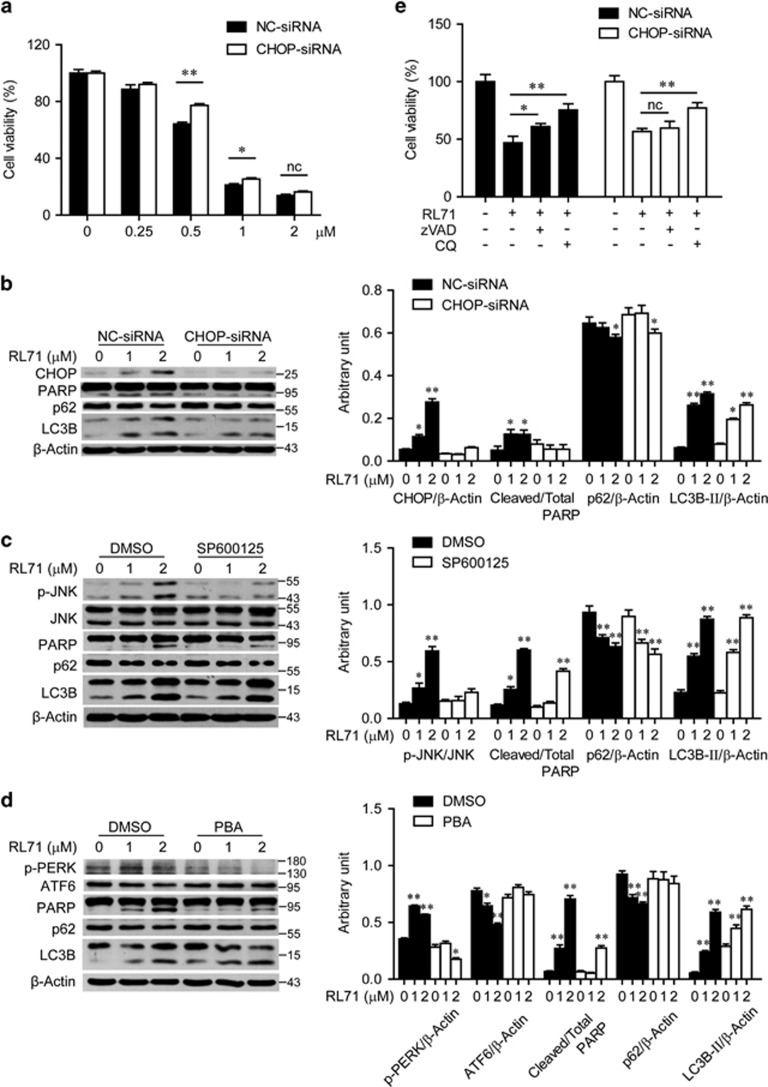
ER stress is responsible for apoptosis, but not for autophagy induced by RL71. (**a**, **b**, **e**) MDA-MB-468 cells were transfected with NC-siRNA or siRNA targeting CHOP for 24 h. (**a**) Cells were treated with various concentrations of RL71 for another 48 h. Cell viability was determined by MTT assay. The data are the mean±S.D. of three independent experiments. *P<0.05, **P<0.01. (**b**) Cells were treated with various concentrations of RL71 for another 24 h. The protein levels of CHOP, PARP, p62 and LC3B were analyzed using western blot. (**c**, **d**) MDA-MB-468 cells were pretreated with (**c**) SP600125 (10 *μ*M) or (**d**) PBA (2.5 mM) for 2 h before treatment with RL71 for 24 h. The indicated protein levels were analyzed. *β*-Actin was used as a loading control. *P<0.05, **P<0.01 *versus* untreated controls. (**e**) Cells with transfection were pretreated with zVAD (20 *μ*M) or CQ (20 *μ*M) for 2 h before treatment with RL71 (1 *μ*M) for 24 h. Cell viability was determined by MTT assay. The data are the mean±S.D. of three independent experiments. *P<0.05, **P<0.01

Considering that a strong and sustained ER stress can lead to cell death by apoptosis and sometimes autophagy, we investigated whether the ER stress induced by RL71 is responsible for apoptosis, autophagy, and finally TNBC cell death. CHOP, a key player in ER stress, was transiently knocked down by using siRNA in MDA-MB-468 cells. Interestingly, siRNA against CHOP reversed cell death induced by low concentrations of RL71 (0.5 and 1 *μ*M), but not by a high concentration (2 *μ*M) (Figure 4a). Knockdown of CHOP abolished the effect of RL71 on PARP cleavage, whereas not inhibiting the reduction of p62 and the conversion of LC3B-I to LC3B-II (Figure 4b). Similar results were obtained in the cells treated with the JNK inhibitor SP600125 or PBA, a known ER stress inhibitor (Figures 4c and d). In addition, we blocked apoptosis and autophagy by zVAD and CQ, respectively. Both zVAD and CQ prevented the decrease in cell viability induced by RL71 in the MDA-MB-468 cells transfected with control siRNA, while only CQ dramatically reduced cell viability in the treated cells with CHOP siRNA (Figure 4e). Taken together, these results suggested that ER stress is not responsible for RL71-induced autophagy.

### Calcium signaling is involved in RL71-induced autophagy

**Figure 5:**
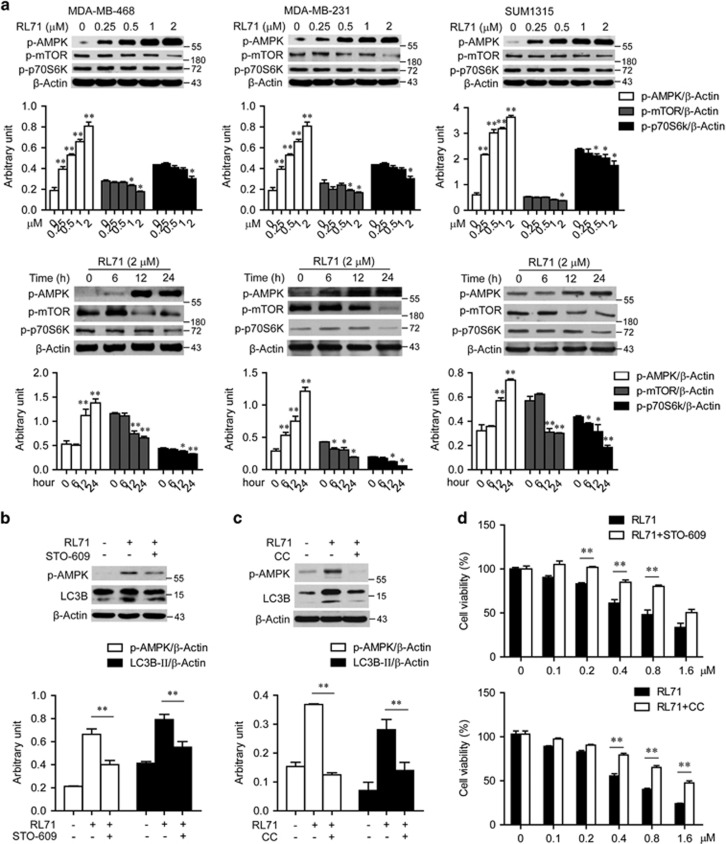
RL71 activates CaMKK-AMPK-mTOR signaling pathway. (**a**) Indicated TNBC cell lines were incubated with various concentrations of RL71 for 24 h or in the presence of RL71 (2 *μ*M) for different time courses. The protein levels of p-AMPK, p-mTOR and p-p70S6K were determined by western blot. *β*-Actin was used as a loading control. *P<0.05, **P<0.01 versus untreated controls. (**b**, **c**) MDA-MB-468 cells were treated with RL71 (2 *μ*M) in the absence or presence of (**b**) STO-609 (25 *μ*M) and (**c**) compound C (CC, 10 *μ*M) for 24 h, respectively. The protein levels of p-AMPK and LC3B were analyzed using western blot. **P<0.01. (**d**) MDA-MB-468 cells were pretreated with STO-609 (25 *μ*M, upper) or CC (10 *μ*M, lower) for 2 h before treatment with various concentrations of RL71 for 24 h. Cell viability was determined by MTT assay. The data are the mean±S.D. of three independent experiments. **P<0.01

A release of Ca^2+^ from the ER into the cytosol can induce autophagy depending on the Ca^2+^/calmodulin-dependent kinase kinase-*β*-dependent activation of AMPK that ultimately leads to the inhibition of mTOR as judged by decreased phosphorylation of the mTOR substrate p70S6K.^18, 19^ As shown in Figure 5a, AMPK phosphorylation was increased by RL71 in dose- and time-dependent manner in all three TNBC cell lines, which was accompanied by a reduction in both mTOR and p70S6K phosphorylation. When MDA-MB-468 cells were treated with RL71 in the presence of the CaMKK inhibitor STO-609, the increase in both AMPK phosphorylation and the conversion of LC3B-I to LC3B-II by RL71 was ameliorated (Figure 5b), supporting the role of CaMKK in RL71-induced autophagy. Also, a striking reduction of the conversion of LC3B-I to LC3B-II was observed in the MDA-MB-468 cells treated with the AMPK inhibitor, compound C (Figure 5c). Cell viability assay revealed that both STO-609 and compound C significantly reversed RL71-induced cell death in MDA-MB-468 cells (Figure 5d). BAPTA, a specific chelator of Ca^2+^, significantly inhibited RL71-induced cell death (Supplementary Figure S3).

**Figure 6:**
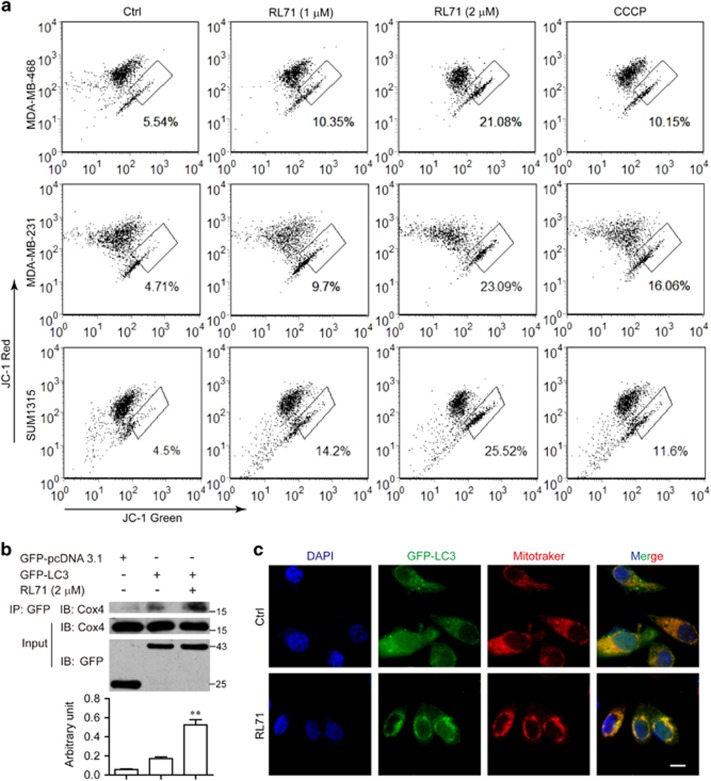
RL71 induces autophagy from the mitochondria. (**a**) Indicated TNBC cell lines were incubated with various concentrations of RL71 for 24 h or CCCP (1 *μ*M) for 1 h. The mitochondrial membrane potential was determined by JC-1 staining and detected using flow cytometry. (**b**) MDA-MB-468 cells transfected with GFP-pcDNA3.1 or GFP-LC3 cDNA were treated with or without RL71 (2 *μ*M) for 24 h. The interaction between GFP-LC3 and Cox4 was measured by coimmunoprecipitation assay. (**c**) MDA-MB-468 cells transfected with GFP-LC3 cDNA were treated with 2 *μ*M of RL71 for 6 h and stained with Mitotracker. Confocal microscopy was performed after a 30-min incubation. Scale bar: 5 *μ*m

Many studies have shown that Ca^2+^-mobilizing agents, including SERCA inhibitors, also can promote mitochondrial membrane depolarization through mitochondrial Ca^2+^ overload.^8, 20^ We thereby investigated whether RL71 caused mitochondrial damage, which promoted the removal of the damaged mitochondria via autophagy. As shown in Figure 6a, RL71 treatment induced mitochondrial potential collapse in dose-dependent manner in all three TNBC cell lines, as demonstrated by a loss of red fluorescence and an increase in green fluorescence of JC-1. Of note, RL71 enhanced the interaction between GFP-LC3 and mitochondrial marker Cox4 in the treated MDA-MB-468 cells overexpressing GFP-LC3 (Figure 6b). Furthermore, we observed a clear co-localization between GFP-LC3 and Mito tracker in MDA-MB-468 cells in response to RL71 (Figure 6c), indicative of autophagic removal of mitochondria.

### RL71 inhibits TNBC tumor development in mice

**Figure 7:**
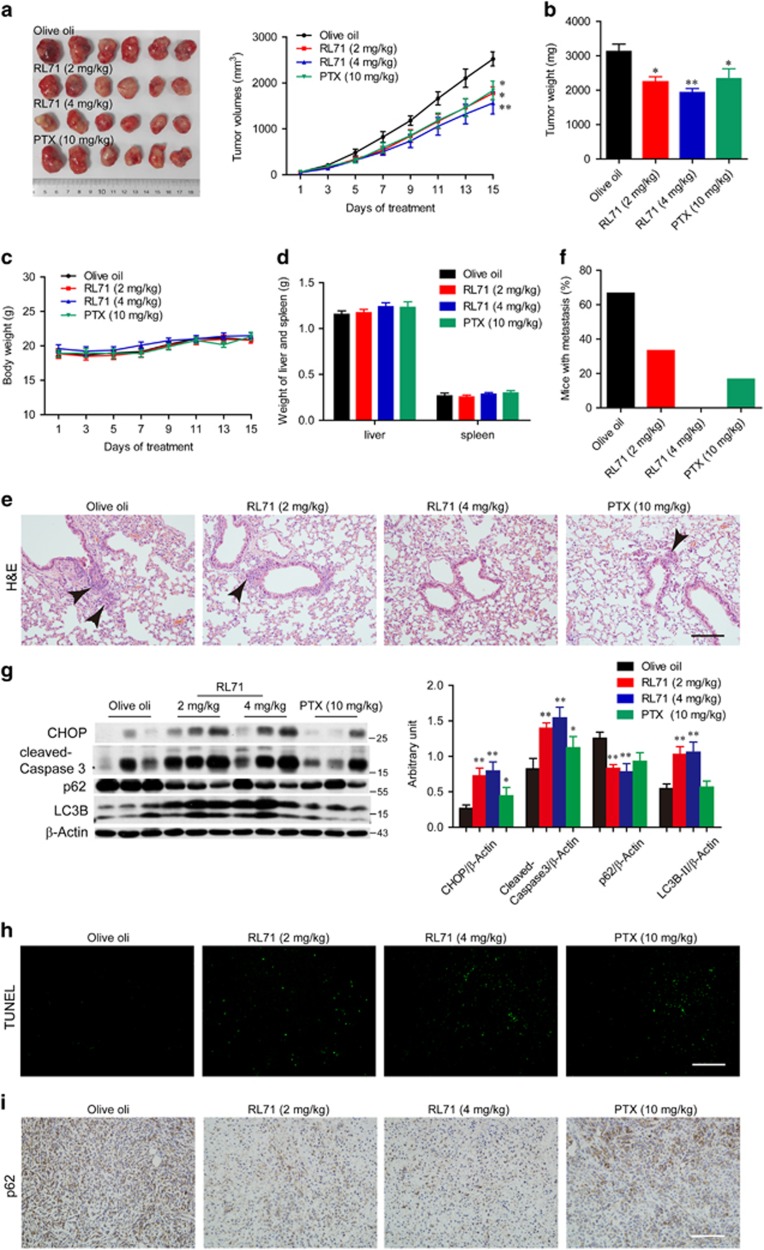
RL71 prevents tumor development in MDA-MB-231 orthotopic inoculation model. Female nude mice were inoculated with 2 × 10^6^ MDA-MB-231 cells into the mammary fat pad. After 14 days, mice (*n*=8 in each group) were treated with olive oil, RL71 (2 or 4 mg/kg/day) or PTX (10 mg/kg/7 days) intraperitoneally for an additional 14 days. (**a**) The tumor growth curves were determined by measuring the tumor volumes. Left panel: representative images of the tumors at the end of the experiment. The data are the mean±S.D. of eight mice per group. *P<0.05, **P<0.01 *versus* the olive oil controls. (**b**) Tumors excised on day 15 were weighed. *P<0.05, **P<0.01 *versus* the olive oil controls. (**c**) The body weight was monitored during the experiment. (**d**) Liver and spleen excised on day 15 were weighed. (**e**) Representative images of lung section stained with H&E. The lung tissues were excised on day 15. Black arrow, metastatic foci in the lungs. Scale bar: 100 *μ*m. (**f**) The proportion of mice with lung metastases in each group. (**g**) Protein levels of CHOP, caspase-3, p62 and LC3B in tumor samples. The tumor tissues were excised on day 15 and analyzed by western blot. *P<0.05, **P<0.01 *versus* the olive oil controls. (**h**) Tumor sections were assayed by TUNEL or (**i**) stained using p62 antibody. The tumor tissues were excised on day 15. The data shown are representative of three experiments. Scale bar: 100 *μ*m

To assess a potential therapeutic effect of RL71 *in vivo*, MDA-MB-231 cells (2 × 10^6^) were inoculated into the mammary fat pad of female athymic nude mice that were treated 14 days later by intraperitoneal injection of olive oil, RL71 (2 or 4 mg/kg/day) or paclitaxel (PTX, 10 mg/kg/7 days) over a period of 2 weeks. Olive oil control mice rapidly developed visible tumors, and we observed dramatic tumor growth throughout the study (Figure 7a). In contrast, RL71 treatment attenuated the development of tumors in a dose-dependent manner. A total of 2 mg/kg of RL71 inhibited the tumor growth to the level comparable to 10 mg/kg of standard-of-care agent PTX. When the tumors were removed on day 15, the average tumor weight was significantly lighter in the mice treated with either RL71 or PTX than the olive oil controls (Figure 7b). Moreover, RL71 treatment induced no apparent toxicity and we noted no change in their behavior, body weight, liver or spleen mass (Figures 7c and d). We further investigated the effect of RL71 on the metastatic property of MDA-MB-231 cells *in vivo*. H&E staining showed extensive pulmonary metastasis in olive oil control mice (Figure 7e). Over 65% of the control mice developed lung metastases (Figure 7f). In contrast, RL71 treatment markedly reduced the incidence of metastasis. No metastatic foci were found in the lungs from the mice treated with 4 mg/kg of RL71, suggesting its great anti-metastatic potential. To investigate the molecular mechanisms involved in the anti-tumorigenic effects of RL71 *in vivo*, tumor tissues excised on day 15 were analyzed. Ca^2+^-ATPase activity in the tumors was inhibited by RL71 in a dose-dependent manner (Supplementary Figure S4a), confirming SERCA2 inhibition *in vivo*. An increase in CHOP expression and caspase-3 cleavage was detected by western blot in the tumors from RL71-treated mice compared with those from the mice treated with olive oil or PTX (Figure 7g). Reduced p62 and increased LC3B-II expression was also shown in the RL71-treated tumors. Terminal deoxynucleotidyl transferase dUTP nick end labeling (TUNEL) assay confirmed the increased apoptosis in RL71-treated tumors (Figure 7h). Furthermore, positive immunostaining for p62 revealed that the RL71-treated tumors had a substantial decrease in p62-positive cells compared with the controls (Figure 7i). The evaluation of both apoptosis by TUNEL assay and autophagy by immunohistochemistry showed a dose-dependent increase in the mice treated with RL71, suggesting their contributions to the anti-tumorigenic effects of RL71.

**Figure 8:**
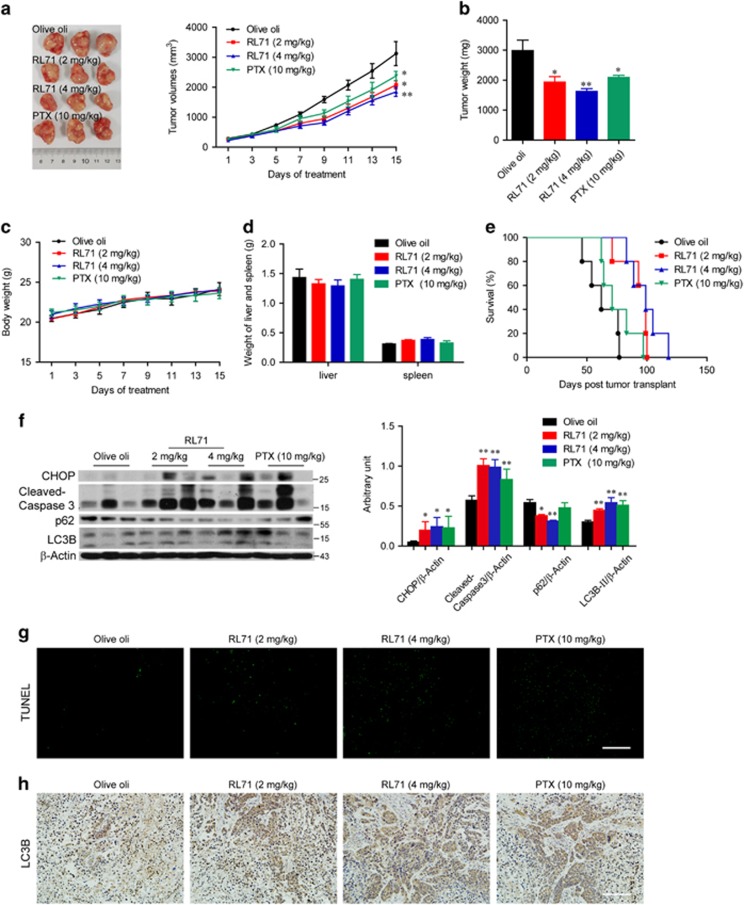
RL71 prevents tumor development in SUM-1315 xenograft model. Female nude mice were inoculated with 5 × 10^6^ SUM-1315 cells into the right flank. After 7 days, mice (*n*=8 in each group) were treated as described in Figure 7. The tumor tissues were excised on day 15 (**a**) The tumor growth curves. Left panel: representative images of the tumors at endpoint. The data are the mean±S.D. of eight mice per group. *P<0.05 *versus* the olive oil controls. (**b**) Tumor weight. *P<0.05, **P<0.01 *versus* the olive oil controls. (**c**) Body weight. (**d**) Weight of liver and spleen on day 15. (**e**) Survival curve of mice (*n*=6 mice per group). (**f**) Protein levels of CHOP, caspase-3, p62 and LC3B in tumor samples. *P<0.05, **P<0.01 *versus* the olive oil controls. (**g**) Tumor sections were assayed by TUNEL or (**h**) stained using LC3 antibody. The data shown are representative of three experiments. Scale bar: 100 *μ*m

To address whether the *in vivo* anti-tumorigenic effects of RL71 are specific to MDA-MB-231 cells, another basal TNBC cell line SUM-1315 (5 × 10^6^) were injected subcutaneously into the right flank of nude mice that were treated as the same as MDA-MB-231 orthotopic inoculation model. Similarly, the reduction in tumor volume and weight was observed in the RL71-treated mice compared with the olive oil controls (Figures 8a and b). RL71 did not have significant effect on the body weight (Figure 8c), as well as the weight of the liver or spleen (Figure 8d). More importantly, the overall survival of the tumor-bearing mice with RL71 treatment was prolonged compared with that of the mice treated with olive oil or PTX (Figure 8e). SERCA2 inhibition *in vivo* was confirmed (Supplementary Figure S4b). Similar results were found concerning induction of apoptosis and autophagy in tumor tissues determined by western blot (Figure 8f). The increased apoptosis and autophagy was confirmed by TUNEL assay and LC3B staining, respectively (Figures 8g and h).

## Discussion

Cytotoxic chemotherapy remains the mainstay of the treatment for TNBC based on the data from many studies over the past two decades.^1^ Despite optimal systemic chemotherapy, virtually all woman with metastatic TNBC will ultimately die of their disease.^21^ The discovery of therapeutic compounds for treating TNBC, especially at the advanced stages, is paramount to further improve patient outcome. Small-molecule RL71 has been reported to show superior cytotoxicity in TNBC cell lines.^12, 13, 14^ However, the underlying mechanisms are still incompletely understood. In the present study, we demonstrate that RL71 displayed potent cytotoxicity on three different TNBC cell lines. RL71-triggered autophagic cell death is responsible for its strong anti-TNBC properties to a larger extent than apoptosis induction. Mechanistically, RL71 augmented the release of Ca^2+^ from the ER into the cytosol by targeting SERCA2. On one hand, the depletion of the ER calcium storage pool induced ER stress leading to apoptosis. On the other hand, a marked increase in cytosolic calcium level triggered autophagic cell death through the activation of intracellular calcium signaling. More importantly, RL71 displayed strong *in vivo* anti-TNBC efficacy including the reduction of metastasis and the prolongation of survival time without obvious toxicity.

A growing body of evidence has revealed both pro- and antitumor role of autophagy in cancer and its therapy.^22, 23^ Many compounds that trigger this biologic response are considered as promising candidates, as exemplified by mTOR inhibitors.^24^ By contrast, there are numerous examples of how autophagy is related to tumor protective effects.^25, 26^ Drugs like CQ are currently used deliberately to inhibit autophagy in ongoing clinical trials. The opposing functions of autophagy may depend on the context.^6, 23^ Extensive autophagy was examined in the context of RL71 treatment for TNBC cells, as evidenced by increased LC3B-I to LC3B-II-conversion, decreased p62 protein expression, as well as accumulation of autophagic vacuoles. RL71 required autophagy to be induced to maximize inhibition of TNBC cell survival, because either silencing *ATG5* or *ATG7*, or pharmaceutically inhibiting autophagy led to a remarkable increase in cell viability. Autophagy variation in the tumor cell determines the likelihood of cell living or dying and thus affecting the success or failure of a therapy intended to reduce tumor growth.^23, 27^ It has been reported that expression of LC3A, LC3B and Beclin-1 was the highest in TNBC cells among the primary breast cancer cells from patients, indicative of high basal autophagy.^11^ It is possible that high levels of basal autophagy in TNBC cells would presumably make induction of excessive autophagic cell death easier. Consistent with this finding, a recent study demonstrates that a small-molecule activator of ULK1, known to be required to initiate the autophagy process, exerts anti-TNBC activity by inducing autophagy-associated cell death.^28^ Given that targeting of cancer stem cells (CSCs) is crucial for the effectiveness of the therapy,^29, 30^ the effect of RL71 on the expression of stemness markers CD44^+^/CD24^−/low^ in TNBC was also tested. However, the subpopulation with the CD44^+^/CD24^−/low^ phenotype failed to be changed significantly in all three cell lines following RL71 treatment (data not shown). It has been reported that CD44^+^/CD24^−^^/low^ CSC has negative LC3 expression in TNBC and LC3^−^/CD44^+^/CD24^−^^/low^ phenotypes are associated with a high risk of poor outcome in TNBC patients.^30^ Possibly, lack of LC3 expression would be responsible for the failure of RL71 action on CSCs in TNBC.

Our previous study identified SERCA2 as the target of RL71. Indeed, RL71 inhibited the Ca^2+^-ATPase activity and induced a steady rise in cytosolic Ca^2+^ levels in MDA-MB-468 cells. Furthermore, RL71 showed a clear co-localization with ER Tracker. Similar results were found in other two TNBC cell lines (data not shown). SERCA has been identified as a therapeutic target for various cancers.^31, 32^ SERCA inhibitors such as TG have been shown to induce autophagic cell death due to the disruption of calcium homeostasis.^8, 33^ As expected, RL71 induced ER stress as supported by the accumulation of unfolded proteins and an elevation of Grp78, ATF4 and CHOP. Although ER stress affects autophagy-related genes, which are evolutionarily conserved and indispensable for autophagy in many cell systems, RL71-induced ER stress was responsible for apoptosis, but not for autophagy in TNBC cells. Inhibiting ER stress including knockdown of CHOP, blockade of JNK activation and addition of the chemical chaperone PBA failed to prevent autophagy induced by RL71. Thus, RL71 targeting SERCA2 induced autophagy independent of ER stress.

Calcium signaling stimulates autophagy by several mechanisms.^34^ In this study, we found that RL71 treatment induced calcium mobilization, leading to the calcium-dependent activation of AMPK, which inhibited activity of the mTOR, a negative regulator of autophagy. Pharmaceutical inhibition of either CaMKK by STO-609 or AMPK by compound C reduced the conversion of LC3B-I to LC3B-II and reversed RL71-induced cell death in MDA-MB-468 cells. In addition, RL71 induced mitochondrial potential collapse in all three TNBC cell lines, which is likely to be due to mitochondrial Ca^2+^ overload. Consistently, many SERCA inhibitors have been shown to induce mitochondrial damage by Ca^2+^ overload.^8, 20^ Although mitoptosis has been also reported relevant with loss of mitochondrial membrane potential,^35^ the formation of mitoptotic bodies was not obviously detected in the treated cells under electron microscopy. RL71-induced mitochondrial damage further potentiated autophagy induction, as evidenced by the increase both in the interaction between GFP-LC3 and Cox4 and in the co-localization between GFP-LC3 and Mito tracker following RL71 treatment. Therefore, the activation of intracellular calcium signaling is essential for RL71-induced autophagy. Considering that the accumulation of autophagic vaculos started at 12 h, whereas <10% apoptotic cells appeared after 24 h of RL71 treatment, we reasoned that calcium signaling-driven autophagy may occur prior to ER stress-mediated apoptosis after RL71 treatment.

TNBC patients do not benefit from hormonal or trastuzmab-based therapy because of the loss of the target receptors such estrogen receptor.^36^ Meanwhile, systemic cytotoxic chemotherapy has major drawbacks including toxicological side effects and drug resistance.^37^ These factors make options for TNBC particularly problematic. In this study, small-molecule RL71 targeting SERCA2 showed strong anti-cancer activity on TNBC cells mainly by triggering excessive autophagy both *in vitro* and *in vivo*. The findings suggest SERCA2 as a novel therapeutic target candidate for TNBC and support the idea that autophagy inducers represent a new approach to treat TNBC.

## Materials and methods

### Reagents and antibodies

RL71 (>97% purity, HPLC) was synthesized as described previously.^14^ Anti-LC3B, anti-ubiqutin, anti-CHOP, anti-PARP, anti-p-JNK, anti-JNK, anti-p-PERK (Thr980), anti-p-AMPK (Thr172), anti-p-mTOR (Ser2448, 2971), anti-p-p70S6K (Thr389), anti-Cox4 and anti-cleaved caspase-3 antibodies were purchased from Cell Signaling Technology (Beverly, MA, USA). Anti-Grp78, anti-ATF4, anti-ATF6, anti-*β*-actin and anti-GFP antibodies were from Santa Cruz Biotechnology (Santa Cruz, CA, USA). Anti-SQSTM1/p62, anti-ATG5 and anti-ATG7 antibodies were from Abcam (Cambridge, UK) and anti-LC3 was from Novus Biologicals (Littleton, CO, USA). GFP-LC3 plasmid was purchased from Yingrun Biotechnologies Inc. (Changsha, China). Compound C (CC) and BAPTA were obtained from Calbiochem (San Diego, CA, USA). zVAD was from Selleck Chemicals (Houston, TX, USA). Chloroquine (CQ), STO-609, SP600125 and PBA were purchased from Sigma-Aldrich (St. Louis, MO, USA). The ER-specific dye ER tracker Red, the mitochondrial specific dye MitoTraker Red CMXRos (M7512), JC-1 (T-3168), Fura-2/AM and Lipofectamine 3000 transfection reagent were purchased from Life Technologies (Grand Island, NY, USA). The Ca^2+^-ATPase activity assay kit was purchased from Nanjing Jiancheng Bioengineering Institute (Nanjing). All of the other chemicals were purchased from Sigma-Aldrich.

### Cell culture

Human TNBC cell lines MDA-MB-231, MDA-MB-468 and SUM-1315 were obtained from the Shanghai Institute of Cell Biology (Shanghai, China). All of the cells were cultured in DMEM medium (Life Technologies) supplemented with 10% fetal bovine serum (Life Technologies), 100 U/ml penicillin and 100 mg/ml streptomycin and incubated at 37 °C in a humidified atmosphere containing 5% CO_2_.

### Animals

Eight-week-old NCR-nu/nu (nude) female mice were purchased from the Model Animal Research Center of Nanjing University (Nanjing). Animal care was performed in compliance with the guidelines of the Ministry of Science and Technology of China (2006) and the related ethical regulations of Nanjing University. All efforts were made to minimize animal suffering and the number of animals used.

### Autophagy analysis

The morphological features of the autophagic cells were assessed using transmission electron microscopy assay as described previously.^38^ For the LC3 assay, MDA-MB-468 cells transfected with GFP-LC3 plasmids were treated with 2 *μ*M RL71 for 12 or 24 h. Then cells were fixed with 4% paraformaldehyde (40 min, room temperature) and permeabilized with methanol and nuclei were stained with DAPI. The formation of vacuoles containing GFP-LC3 (dots) was examined by fluorescence microscopy (BX51TRF, Olympus Corporation, Shinjuku, Tokyo).

### Western blot and coimmunoprecipitation assay

The protocols for western blot and coimmunoprecipitation have been reported previously.^38^ The densitometry of immunoblots was quantified with Image J software (NIH, Bethesda, MD, USA).

### RNA interference

Chemically synthesized sense and anti-sense RNA oligonucleotides were obtained from Guangzhou RiboBio Co., Ltd. (Guangzhou, China). ATG5 siRNA sequences were 5′-GUGAGAUAUGGUUUGAATA-3′. ATG7 siRNA sequences were 5′-CCAACACACUCGAGUCUUU-3′. CHOP siRNA sequences were 5′-UUGAGCCGUUCAUUCUCUUCAGCUA-3′. SERCA2 siRNA sequences were 5′-CAAAGUUCCUGCUGAUAUA-3′. Luciferase siRNA was used as described previously.^38^ Cells were plated on six-well plates at 5 × 10^5^ cells per well and transfected with 50–100 pmol of siRNA using Lipofectamine 3000.

### Intracellular Ca^2+^ measurement

[Ca^2+^]_i_ was determined using the Ca^2+^-sensitive fluorescent indicator Fura-2/AM as previously described.^14^

### Confocal microscopy

MDA-MB-468 cells were incubated with RL71 for 2 h and then fixed in 4% paraformaldehyde (pH 7.4) for 10 min at 37 °C. The co-localization of RL71 and ER tracker or of LC3-GFP and MitoTracker was analyzed following the manufacturer’s protocol. The fluorescent signals were detected using a FluoViewTM FV1000 confocal microscope (Olympus) and analyzed by the Olympus Fluview Ver1.7b viewer (Olympus).

### Mitochondrial membrane potential assay

The disruption of mitochondrial membrane potential was measured using JC-1 staining by flow cytometry as previously described.^39, 40^ Briefly, cells were incubated with various concentrations of RL71 for 24 h. A potent mitochondrial uncoupling agent CCCP was used as a positive control. Cells were stained with 5 *μ*g/ml of JC-1 for 20 min at 37 °C and then analyzed for the decrease in red–orange fluorescence using a FACSCalibur flow cytometer (Becton Dickinson, San Jose, CA, USA).

### *In vivo* murine cancer models

Two murine cancer models were used to examine the effectiveness of RL71. Breast cancer cell engraftment to the mammary fat pad of mice better recapitulates the location of the disease and presence of the proper stromal compartment and therefore better mimics human cancerous disease.^41^ Our pilot experiment showed that MDA-MB-231 cells were optimal for orthotopic growth in the mice despite their growing at a slow rate. In this study, MDA-MB-231 cells (2 × 10^6^ cells in 20 *μ*l PBS) were subcutaneously injected near the fat pad of the fourth mammary gland in the lower abdomen of nude mice. Two weeks after the injection, the mice bearing tumors (an average size of 50 mm^3^) were distributed into four groups (*n*=8 mice per group). Olive oil, RL71 (2 or 4 mg/kg/day) or Paclitaxel (PTX, 10 mg/kg/7 days) were administered for 14 days by intraperitoneal injection. For another, a widely used model that is the subcutaneous injection of breast cancer cells into mice was established to monitor the survival rate. SUM-1315 cells (5 × 10^6^ cells in 0.1 ml PBS) with great metastatic potential were injected into the right flank of nude mice. One week after the injection, the mice were treated as described as above. Tumor volumes were measured every 2 days and calculated using the following formula: 0.5236 × *L1* × (*L2*)^2^, where *L1* and *L2* are the long and short diameters of the tumor mass, respectively. Tumor tissues, lung, liver and spleen were excised on day 15. In SUM-1315 xenograft model, survival test was made (*n*=6 mice per group) as above and monitored daily until all the mice died.

### Histologic analysis, TUNEL assay and immunohistochemistry

Lung sections from mice were obtained for H&E staining and analyzed by a pathologist using a light microscope (Olypus). TUNEL assay was performed using *In Situ* Cell Death Detection Kit (Roche, Basel, Switzerland). Immunostaining of LC3B was performed using a Real Envision Detection kit from the Gene Tech Company (Shanghai) according to the manufacturer’s instructions.

### Statistical analysis

Data are expressed as the means±S.D. The Student’s *t*-test was used to evaluate the difference between groups. In some experiments, statistical analyses were performed using one-way analysis of variance followed by a *post hoc* test. The Kaplan–Meier method was used to evaluate the survival results. *P*<0.05 was considered significant. All the data were generated from at least three independent experiments. All statistical analyses were conducted using SPSS version 10.0 statistical software (SPSS, Chicago, IL, USA).

## Publisher’s Note:

Springer Nature remains neutral with regard to jurisdictional claims in published maps and institutional affiliations.
