# Supplementary material for: Small-molecule RL71-triggered excessive autophagic cell death as a potential therapeutic strategy in triple-negative breast cancer
Source: Cell Death Dis. 2017 Sep 14;8(9):e3049–. doi: 10.1038/cddis.2017.444 (PMC5636988; doi:10.1038/cddis.2017.444)
Supplement: Supplementary Information [file cddis2017444x1.pdf]

Small molecule RL71-triggered excessive autophagic cell death as a potential therapeutic strategy in triple-negative breast cancer

Jian Gao[1,4], Minmin Fan[1,4], Shuang Peng[1], Minxia Zhang [1], Gang Xiang[1], Xin Li[1], Wenjie Guo[1], Yang Sun[1], Xuefeng Wu[1], Xudong Wu[1], Guang Liang[2], Yan Shen[*,1], Qiang Xu[*,1,3]

Supplementary method

Cytotoxicity assay. Cell viability was detected using MTT assay. Cell death rate was assessed using the Trypan Blue dye exclusion method with manual cell counting using a hemocytometer (XB.K.25, QIUJING, Shanghai). To test the cytotoxic effect of RL71 on cancer stem cells, the expression of stemness markers $CD44^{+}/CD24^{-/low}$ in TNBC was analyzed using a FACSCalibur flow cytometer (Becton Dickinson, San Jose, CA). Fluorescently labeled anti-CD44 and anti-CD24 antibodies were from BD Pharmingen (San Jose, CA).

Apoptosis analysis. Cell apoptosis was determined by Hoechst staining or by flow cytometry after addition of FITC-conjugated annexin V and PI. Annexin V^{+}/PI^{-} and

annexin V^+/PI^+ were considered apoptotic cells in the early and late phase, respectively. The samples were analyzed by flow cytometry on FACSCalibur flow cytometer.

Supplementary Figures

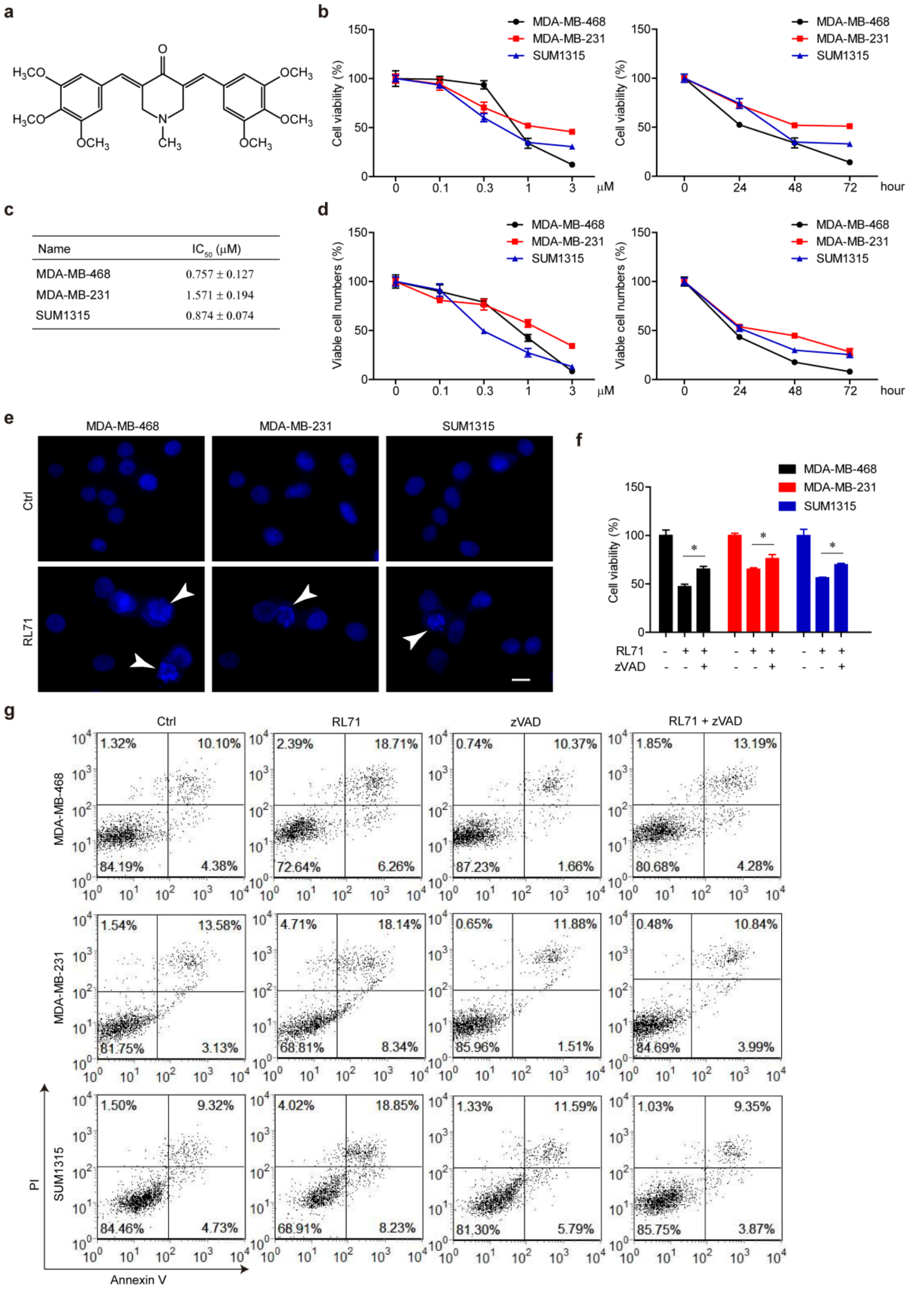

Supplementary Figure S1. RL71 exerts potent cytotoxic activity in TNBC cell lines partly due to apoptosis induction. (a) Chemical structure of RL71. Indicated

TNBC cell lines were incubated with various concentrations of RL71 (0-3 μM) for 48 h or in the presence of RL71 (1 μM) for different time courses. Cell viability was determined by MTT assay (b), and IC_{50} was shown (c). The number of cells was counted using a Trypan Blue exclusion assay (d). The data are the mean ± SD of three independent experiments. (e) Cells were incubated in the absence or presence of RL71 (1 μM) for 24 h and stained with Hoechst staining. White arrow, apoptotic cells with typical chromatins condensation and blebbing nuclei. Scale bar: 10 μm. (f) Cells were pretreated with zVAD (20 μM) for 2 h before treatment with RL71 (1 μM) for 24 h. Cell viability was determined by MTT assay. The data are the mean ± SD of three independent experiments. *P<0.05. (g) Cells were pretreated with zVAD (20 μM) for 2 h before treatment without or with RL71 (1 μM) for 48 h. The percentages of apoptotic cells were determined by annexin V/PI double staining.

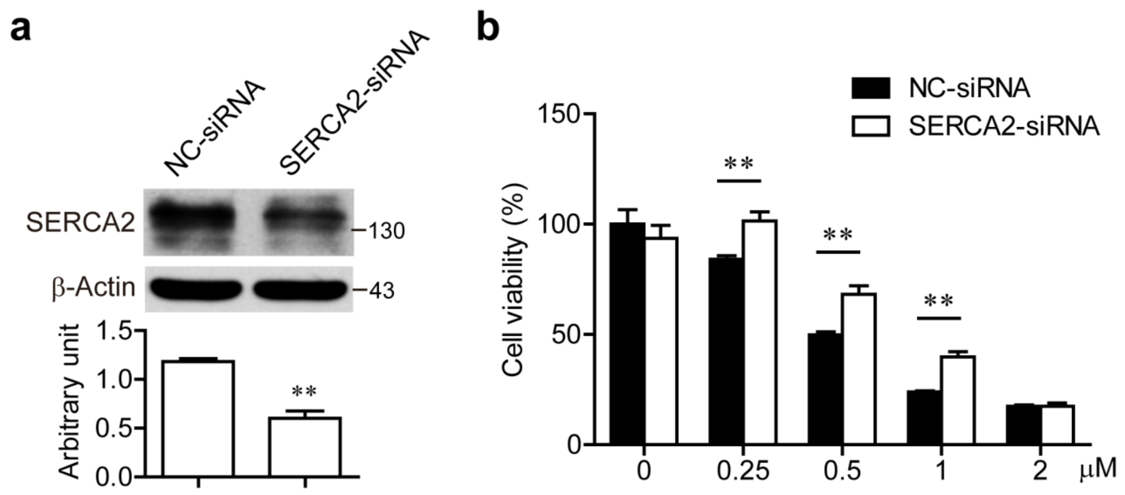

Supplementary Figure S2. Effect of knocking down SERCA2 on cytotoxic activity of RL71. MDA-MB-468 cells were transfected with SERCA2-targeting siRNA. The resulting cells were treated with various concentrations of RL71 for 48 h. (a) Knockdown of SERCA2. (b) Cell viability was determined by MTT assay. The data are expressed as the mean ± SD of three independent experiments. ***P*<0.01.

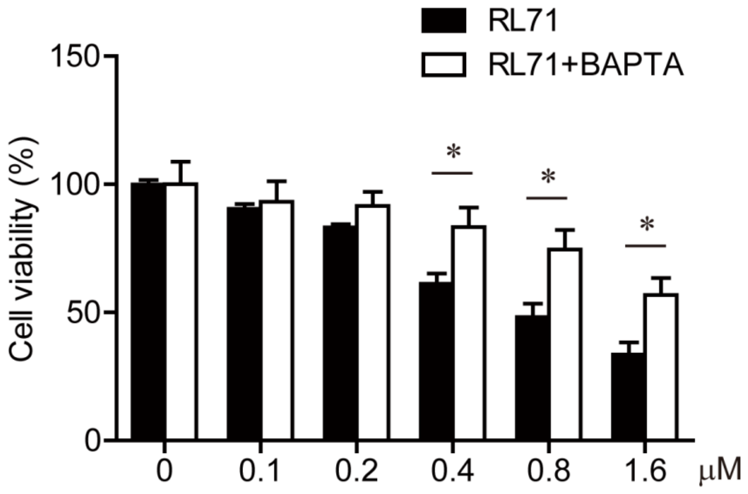

Supplementary Figure S3. Effect of calcium chelator BAPTA on cytotoxic activity of RL71. MDA-MB-468 cells were pretreated with BAPTA (10 μM) for 2 h before treatment with various concentrations of RL71 for 24 h. Cell viability was determined by MTT assay. The data are the mean ± SD of three independent experiments. **P*<0.05.

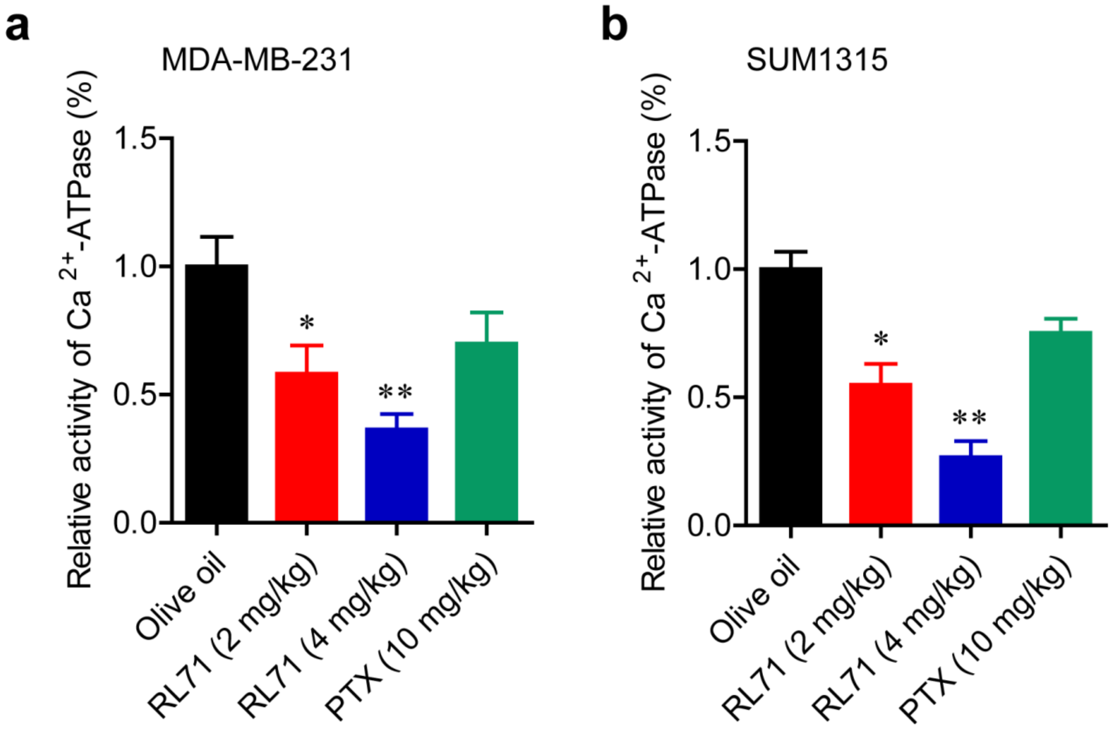

Supplementary Figure S4. Inhibition of Ca^{2+}-ATPase activity by RL71 *in vivo*.

The tumor tissues were excised on day 15 from (a) MDA-MB-231 orthotopic inoculation model and (b) SUM1315 xenograft model, and measured according to the instructions of the Ca^{2+}-ATPase kit.
